# Analysis of Immune Checkpoints on Peripheral Blood Mononuclear Cells Can Predict Clinical Outcome and Reveal Potential of HVEM-BTLA Axis in Epithelial Ovarian Cancers

**DOI:** 10.3390/ph18091295

**Published:** 2025-08-29

**Authors:** Yen-Ling Lai, Han-Wei Lin, Yu-Wen Huang, Jung Chen, Ming-Chien Tai, Chia-Ying Wu, Tyan-Shin Yang, Valentina Oblin, Kristin Shea, Yu-Li Chen

**Affiliations:** 1 Department of Obstetrics and Gynecology, National Taiwan University Hospital Hsin-Chu Branch, Hsin Chu 300195, Taiwan; adina@kimo.com; 2 Department of Obstetrics and Gynecology, National Taiwan University Hospital, College of Medicine, National Taiwan University, Taipei 100233, Taiwan; handway_rc@hotmail.com (H.-W.L.); jjchen915@gmail.com (J.C.); s90091724@yahoo.com.tw (M.-C.T.); ascheetah@msn.com (C.-Y.W.); jessica0617xyz@gmail.com (T.-S.Y.); 3 Department of Medical Research, National Taiwan University Hospital, Taipei 100225, Taiwan; yuwen719@ntuh.gov.tw; 4 Department of Obstetrics and Gynecology, National Taiwan University Hospital Yun-Lin Branch, Yun Lin County 640203, Taiwan; 5 Department of Obstetrics and Gynecology, Division of Obstetrics and Feto-maternal Medicine, Medical University of Vienna, 1090 Vienna, Austria; valentina.oblin@meduniwien.ac.at; 6 Crean College of Health and Behavioral Sciences, Chapman University, Orange, CA 92866, USA; kristin.shea25@gmail.com

**Keywords:** PD-1-expressing T lymphocytes, prognosis, BTLA-HVEM axis, cancer immunotherapy, epithelial ovarian cancer

## Abstract

**Background/Objectives:** Immune checkpoint inhibitors (ICIs) do not provide promising benefits to patients with advanced epithelial ovarian cancer (EOC). This study analyzed preoperative peripheral blood mononuclear cells (PBMCs) from these patients to evaluate the prognostic and therapeutic checkpoints. **Methods:** Preoperative PBMCs of 69 advanced EOC cases were collected to analyze the correlation between IC-expressing immune cells and survivals of patients. Co-expression of various ICs on the T lymphocytes from these patients was examined. Activation potential of programmed cell death 1 (PD-1)^+^herpes virus entry mediator (HVEM)^+^ T cells in PBMCs from the healthy donors and tumoricidal abilities of PMBCs treated with various ICIs were evaluated in vitro. Impact of respective ICIs on activation of T cells in PMBCs was investigated. **Results:** Percentages of PD-1^+^ CD4^+^ and CD8^+^ T cells in the PBMCs of patients could positively correlate with disease-free or overall survival. HVEM was highly co-expressed on these T lymphocytes. Prediction potential for overall survival of patients by the subpopulation of PD-1^+^ CD4^+^ or CD8^+^ T cells was higher than that by other parameters. The PD-1^+^HVEM^+^ CD4+ and CD8^+^ T cells showed characteristics of activated phenotype under activation signals. PBMCs receiving anti-B and T lymphocyte attenuator (BTLA) plus anti-cytotoxic T lymphocyte antigen 4 (CTLA-4) or anti-PD-1 Ab had potent tumor-killing ability. Anti-BTLA Ab can drive T cells in the PBMCs toward an effector status. **Conclusions:** Percentages of PD-1^+^ T cells in the PBMCs could predict survival of EOC patients. Targeting HVEM-BTLA axis may be considered for ICI treatment of EOCs.

## 1. Introduction

Epithelial ovarian cancer (EOC) is the most lethal malignancy of all gynecological cancers [1,2]. Patients are often diagnosed with EOC in advanced stages. The standard treatment approach is based on cytoreductive surgery followed by platinum/paclitaxel (PTX)-based systemic chemotherapy [3]. Following the systemic chemotherapy, anti-angiogenic agent and inhibitors of the poly-ADP ribose polymerase (PARP) are commonly applied in the maintenance treatment [4,5,6,7]. Despite these treatment strategies, the risk of tumor recurrence remains high, highlighting the need for investigation of effective therapies, including cancer immunotherapy.

Immune checkpoint inhibitors (ICIs), one of the immunomodulatory therapies, enhances the activity of host immune system by inhibiting the suppressive signals of checkpoint molecules. These agents can lead to activation and proliferation of tumor-specific T cells, which are able to eliminate tumor cells [8]. Cytotoxic T-lymphocyte antigen 4 (CTLA-4) and programmed death 1 (PD-1) are well-known checkpoints because of their respective ICIs in clinical applications [9,10,11]. High expression of programmed death-ligand 1 (PD-L1) has been reported as a negative prognostic factor in ovarian cancer, and the PD-1/PD-L1 axis can be a target for restoring antitumor immunity [12]. In addition to PD-1 and CTLA-4, other inhibitory checkpoints, such as T-cell immunoglobulin and mucin-domain containing-3 (TIM-3) and the B- and T-lymphocyte attenuator (BTLA)–herpesvirus entry mediator (HVEM) axis, are currently evaluated in clinical trials as potential targets for immunotherapy [13,14]. TIM-3 is expressed on exhausted T cells and other immune cells, where it contributes to immune suppression within the tumor microenvironment [15].

BTLA is an inhibitory checkpoint receptor expressed on T cells, B cells, macrophages, dendritic cells, and natural killer cells. Its primary ligand, HVEM, is broadly expressed and can also bind CD160 or LIGHT (homologous to lymphotoxin, exhibits inducible expression and competes with HSV glycoprotein D for binding to HVEM), although BTLA has higher affinity for HVEM [16]. BTLA–HVEM engagement suppresses T-cell activation, suggesting that its blockade could enhance antitumor immunity, potentially by promoting LIGHT-HVEM signaling [17]. However, monotherapy of ICIs cannot show the advantage over chemotherapy in advanced and recurrent EOCs in the results of current clinical trials [18]. One mechanism of poor therapeutic responses may be the co-expression of multiple immune checkpoints (ICs) on the CD8^+^ T lymphocytes. These T cells have been described as exhausted and associated with tumor progression [19,20,21].

Most previous studies investigated prognostic role or therapeutic potential of ICs in cancer patients using immune components from tumor microenvironment (TME). In this current study, peripheral blood mononuclear cells (PBMCs) of the advanced EOC cases were utilized to analyze the correlation between IC-expressing immune cells and survival outcomes of patients. The status of co-expression of various checkpoint molecules on the T lymphocytes from these EOC cases was further examined by t-distributed stochastic neighbor embedding (t-SNE) algorithm. Finally, the potential role of therapeutic antibodies (Abs) targeting associated IC signaling on the modulation of T lymphocytes was evaluated in vitro using PBMCs from the healthy donors.

## 2. Results

### 2.1. PD-1^+^ T lymphocytes in PBMCs Were Associated with the Survival of EOC Patients

Based on the clinico-pathological characteristics of the study population (Supplementary Table S1), the median age at disease diagnosis was 58.0 years and median follow-up duration was 63.0 months. Most patients had stage III diseases (58/69) and serous carcinomas (61/69). A total of 9 patients underwent neoadjuvant chemotherapy followed by interval cytoreductive surgeries and postoperative chemotherapy and the remaining 60 received interval cytoreductive surgeries followed by postoperative chemotherapy. Largest diameter of postoperative residual tumor ≤ 1 cm could be observed in 49 patients. Overall, 54 of 69 cases had tumor relapse and 22 of 69 died of disease.

**Figure 1:**
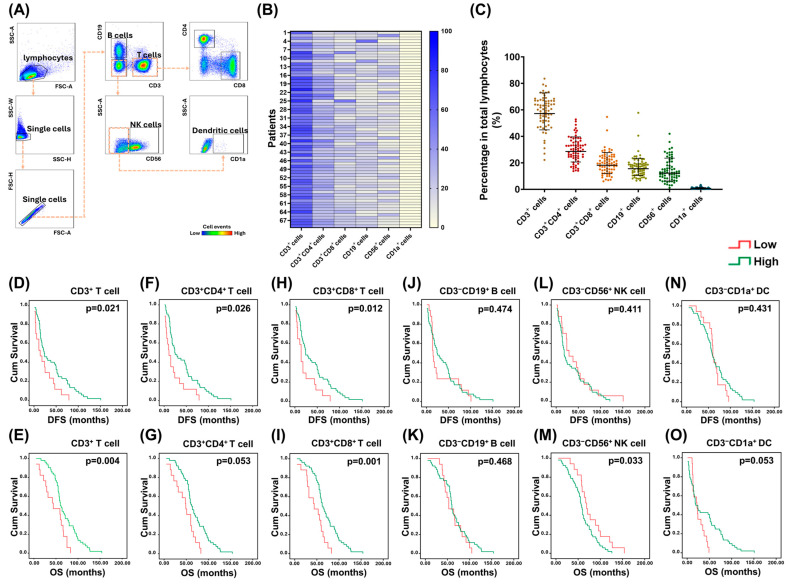
Survival impact of various immunocytes in lymphocytes of PBMCs. (**A**) Representative dot plot from flow cytometry illustrating the gating strategy. (**B**) Heatmap of the expression of various immunocytes in PBMCs from 69 EOC patients. (**C**) Dot plots showing the percentages of various immunocytes in lymphocytes of PBMCs. CD3^+^ T cells (57.2 ± 1.3%) were the most common immunocytes, followed by CD19^+^ B cells (15.6 ± 1.5%), CD56^+^ NK cells (12.2 ± 1.9%), and CD1a^+^ DCs (0.7 ± 1.9%). (**D**–**O**) Kaplan–Meier analyses of the DFS and OS for 69 EOC cases according to the percentages of various immune cells. DFS or OS of the patients with higher percentages of CD3^+^ T cells (DFS: 23.0 ± 6.0 months; OS: 60.0 ± 3.6 months), CD3^+^CD4^+^ T cells (DFS: 23.0 ± 5.2; months; OS: 62.0 ± 4.0 months), or CD3^+^CD8^+^ cells (DFS: 24.0 ± 5.4 months; OS: 61.0 ± 4.1 months) was longer than that of cases with lower percentages of CD3^+^ T lymphocytes (DFS: 14.0 ± 6.2 months; OS: 44.0 ± 18.5 months), CD3^+^CD4^+^ T lymphocytes (DFS: 11.0 ± 3.7 months; OS: 49.0 ± 6.9 months), or CD3^+^CD8^+^ T lymphocytes (DFS: 14.0 ± 2.0 months; OS: 44.0 ± 14.4 months). However, the OS of those patients with higher percentages of CD3^−^CD56^+^ NK cells (57.0 ± 2.5 months) was shorter than that of cases with lower percentages of CD3^−^CD56^+^ NK cells (69.0 ± 4.2 months). Note: (**D**,**E**) CD3^+^ T cells, (**F**,**G**) CD3^+^CD4^+^ T cells, (**H**,**I**) CD3^+^CD8^+^ T cells, (**J**,**K**) CD3^−^CD19^+^ B cells, (**L**,**M**) CD3^−^CD56^+^ NK cells, (**N**,**O**) CD3^−^CD1a^+^ dendritic cells.

A representative dot plot from flow cytometry illustrating the gating strategy and a heatmap of the expression of various immunocytes in PBMCs from 69 EOC patients are shown in Figure 1A,B, respectively. Among these immune cells, CD3^+^ T cells are the most common immunocytes in PBMCs, followed by CD19^+^ B cells, CD56^+^ NK cells, and CD1a^+^ dendritic cells (Figure 1C). Association between the percentages of various immunocytes and the survivals of study population was analyzed (Figure 1D–O). The disease-free survival (DFS) or overall survival (OS) of patients with higher percentages of CD3^+^ T cells, CD3^+^CD4^+^ T cells, or CD3^+^CD8^+^ T cells was longer than that of cases with lower percentages of CD3^+^ T lymphocytes, CD3^+^CD4^+^T lymphocytes, or CD3^+^CD8^+^ T lymphocytes (Figure 1D–I). However, the OS of patients with higher percentages of CD3^−^CD56^+^ NK cells was shorter than that of cases with lower percentages of CD3^−^CD56^+^ NK cells (Figure 1M). CD19^+^ B cell expression did not correlate with patient survival (Figure 1J,K). CD1a^+^ dendritic cell expression did not correlate with patient DFS (Figure 1N). Compared with patients with lower percentages of CD1a^+^ dendritic cell, those with higher percentages of CD1a^+^ DC cells had a trend of longer OS (Figure 1O).

**Figure 2:**
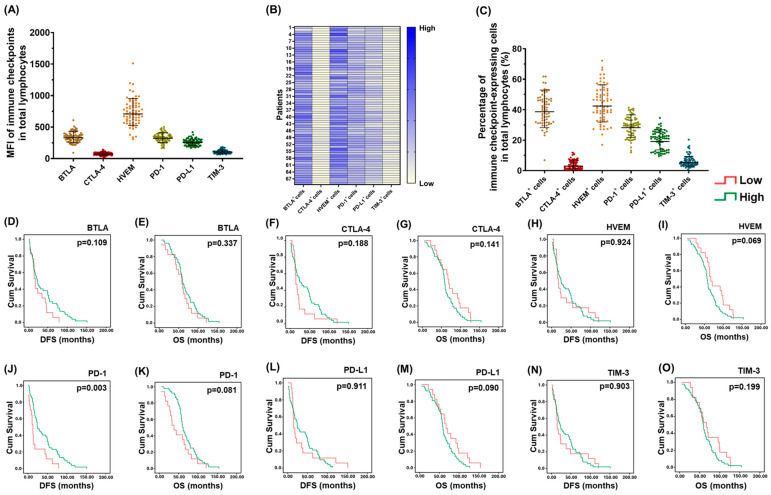
Survival impact of various IC-expressing cells in lymphocytes of PBMCs. (**A**) Dot plots exhibiting the MFIs of various ICs in lymphocytes of PBMCs. MFI of HVEM (708.1 ± 1.3) was the highest in PBMCs, followed by BTLA (329.8 ± 1.3), PD-1 (322.2 ± 1.3), PD-L1 (253.4 ± 1.2), TIM-3 (104.7 ± 1.3), and CTLA-4 (71.6 ± 1.3). (**B**) Heatmap of the expression of various IC-expressing cells in PBMCs from 69 EOC patients. (**C**) Dot plots exhibiting the percentages of various IC-expressing cells in lymphocytes of PBMCs. HVEM^+^ cells (42.4 ± 1.3%) were the most common in PBMCs, followed by BTLA^+^ cells (38.6 ± 1.4%), PD-1^+^ cells (28.2 ± 1.3%), PD-L1^+^ cells (19.1 ± 1.4%), TIM-3^+^ cells (5.3 ± 1.8%), and CTLA-4^+^ cells (2.9 ± 2.4%). (**D**–**O**) Kaplan–Meier analyses of the DFS and OS for 69 EOC cases according to the percentages of various IC-expressing cells. DFS of the patients with higher percentages of PD-1^+^ cells (24.0 ± 4.9 months) was longer than that of cases with lower percentages of PD-1^+^ cells (13.0 ± 2.0 months). Note: (**D**,**E**) BTLA^+^ cells, (**F**,**G**) CTLA-4^+^ cells, (**H**,**I**) HVEM^+^ cells, (**J**,**K**) PD-1^+^ cells, (**L**,**M**) PD-L1^+^ cells, (**N**,**O**) TIM-3^+^ cells.

Various IC expression in the lymphocytes of PBMCs was analyzed and the mean of fluorescence intensity (MFI) is presented in Figure 2A. MFI of HVEM was the highest in PBMCs. A heatmap of the expression of various IC-expressing cells in PBMCs from the study population is shown in Figure 2B. Among the IC-expressing cells, HVEM^+^ cells were the most common in PBMCs, followed by BTLA^+^ cells, PD-1^+^ cells, PD-L1^+^ cells, TIM-3^+^ cells, and CTLA-4^+^ cells (Figure 2C). Association between the percentages of various IC-expressing cells and the survivals of 69 EOC patients was examined (Figure 2D–O). The DFS of patients with higher percentages of PD-1^+^ cells was longer than that of cases with lower percentages of PD-1^+^ cells (Figure 2J). In addition, a trend toward longer OS could be observed in patients with higher percentages of PD-1^+^ cells (Figure 2K).

**Figure 3:**
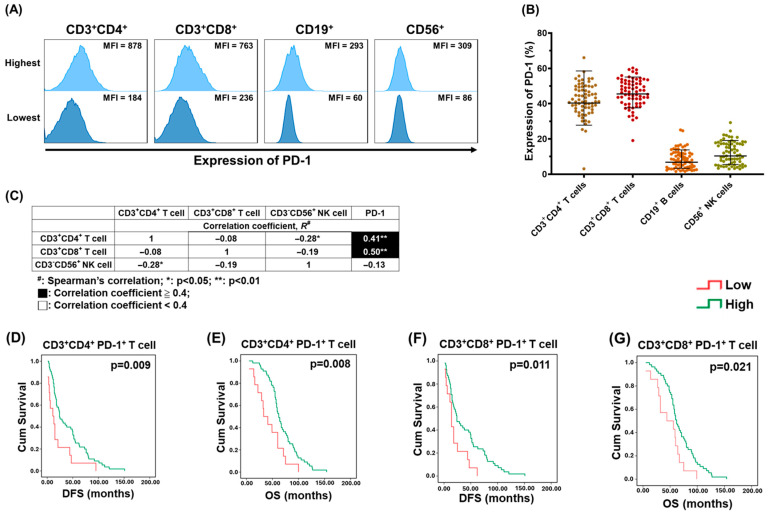
Correlation between various immunocytes and PD-1^+^ cells in lymphocytes of PBMCs. (**A**) Representative histograms showing the range of PD-1 MFI in various immunocytes from 69 EOC patients. (**B**) Dot plots exhibiting the percentage of PD-1-expressing immunocytes in lymphocytes of PBMCs. CD3^+^CD8^+^ T cells (45.5 ± 1.2%) presented highest PD-1 expression, followed by CD3^+^CD4^+^ T cells (40.3 ± 1.5%), CD56^+^ NK cells (10.34 ± 1.9%), and CD19^+^ B cells (6.8 ± 2.0%). (**C**) The correlation between various immunocytes and PD-1-expressing cells in PBMCs from 69 EOC patients. Percentage of CD3^+^CD4^+^ or CD3^+^CD8^+^ T lymphocytes had a high correlation (correlation coefficient, R ≥ 0.4) with that of PD-1-expressing cells. (**D**–**G**) Kaplan–Meier analyses of the DFS and OS for 69 EOC cases according to the level of CD3^+^CD4^+^PD-1^+^ or CD3^+^CD8^+^PD-1^+^ T lymphocytes. DFS or OS of the patients with higher percentages of PD-1^+^CD4^+^ T lymphocytes (DFS: 24.0 ± 4.8 months; OS: 62.0 ± 3.7 months) or PD-1^+^CD8^+^ T lymphocytes (DFS: 24.0 ± 5.3 months; OS: 62.0 ± 4.2 months) was better than that of cases with lower percentages of CD3^+^CD4^+^PD-1^+^ T cells (DFS: 11.0 ± 6.5 months; OS: 33 ± 8.4 months) or CD3^+^CD8^+^PD-1^+^ T cells (DFS: 14.0 ± 1.9 months; OS: 44.0 ± 22.5 months). Note: (**D**,**E**) CD3^+^CD4^+^PD-1^+^ T cells, (**F**,**G**) CD3^+^CD8^+^PD-1^+^ T cells.

Representative histograms of the range of PD-1 MFI in various immunocytes from 69 EOC patients are presented in Figure 3A. Percentages of PD-1 expression in CD3^+^CD4^+^ and CD3^+^CD8^+^ T cells were higher than those in B and NK cells (Figure 3B). The correlation between various immunocytes and PD-1-expressing cells in PBMCs was further evaluated. The percentage of CD3^+^CD4^+^ or CD3^+^CD8^+^ T lymphocytes had a high correlation with that of PD-1-expressing cells (Figure 3C). Association between the percentages of CD3^+^CD4^+^PD-1^+^ or CD3^+^CD8^+^PD-1^+^ cells and the survival of 69 EOC cases was analyzed (Figure 3D–G). Patients with higher percentages of PD-1^+^CD4^+^ T lymphocytes (Figure 3D,E) or PD-1^+^CD8^+^ T lymphocytes (Figure 3F,G) in PBMCs had better survival, including DFS and OS compared to those with lower percentages of CD3^+^CD4^+^PD-1^+^ T cells or CD3^+^CD8^+^PD-1^+^ T cells.

### 2.2. Co-Expression of HVEM on PD-1^+^ T lymphocytes Was Highly Detected

The percentage of CD3^+^CD4^+^PD-1^+^ or CD3^+^CD8^+^PD-1^+^ T cell population in PBMCs could predict the outcome of ovarian cancer in this study. However, the antitumor effect of anti-PD-1 agents in EOC was less satisfactory than expected in the results of clinical trials [18]. One mechanism underlying poor therapeutic responses might be the co-expression of various inhibitory ICs on effector T lymphocytes. These T cells expressing multiple ICs have been reported as exhausted, associated with tumor progression, and can be investigated by the t-SNE algorithm [19,20,21,22].

**Figure 4:**
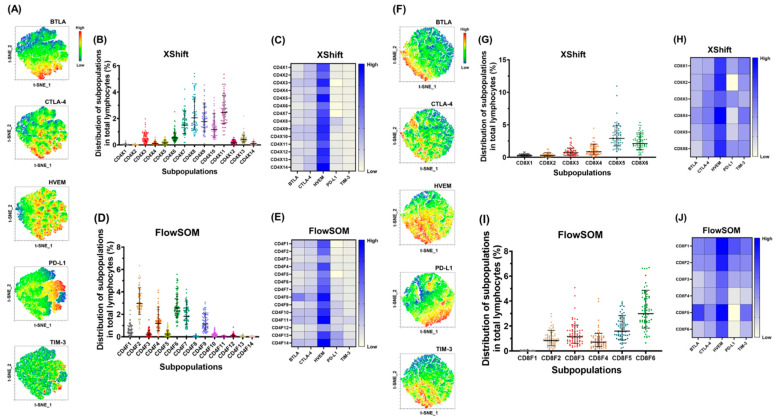
Co-expression of other ICs on CD3^+^CD4^+^PD-1^+^ and CD3^+^CD8^+^PD-1^+^ T cells identified from the t-SNE algorithm. (**A**) Expression of various ICs on CD3^+^CD4^+^PD-1^+^ T cells on the raw t-SNE map. (**B**) Bar figures showing the percentages of 14 CD3^+^CD4^+^PD-1^+^ T cell subpopulations by XShift clustering. Cluster X11 (2.5 ± 1.5%) was the most common subpopulation. (**C**) Heatmap showing the expression level of various ICs in the 14 CD3^+^CD4^+^PD-1^+^ T cell subpopulations by XShift clustering. Among these ICs, HVEM had the highest co-expression level. (**D**) Bar figures showing the percentages of 14 CD3^+^CD4^+^PD-1^+^ T cell subpopulations by FlowSOM clustering. Cluster F2 (3.0 ± 1.5%) was the most common subpopulation. (**E**) Heatmap showing the expression level of various ICs in the 14 CD3^+^CD4^+^PD-1^+^ T cell subpopulations by FlowSOM clustering. Among these ICs, HVEM had the highest co-expression level. (**F**) Expression of immune checkpoints on CD3^+^CD8^+^PD-1^+^ T cells on the raw t-SNE map. (**G**) Bar figures showing the percentages of 6 CD3^+^CD8^+^PD-1^+^ T cell subpopulations by XShift clustering. Cluster X5 (2.9 ± 1.7%) was the most common subpopulation. (**H**) Heatmap showing the expression level of various ICs in the 6 CD3^+^CD8^+^PD-1^+^ T cell subpopulations by XShift clustering. Among these ICs, HVEM had the highest co-expression level. (**I**) Bar figures showing the percentages of 6 CD3^+^CD8^+^PD-1^+^ T cell subpopulations by FlowSOM clustering. Cluster F6 (3.0 ± 1.6%) was the most common subpopulation. (**J**) Heatmap showing the expression level of various ICs in the 6 CD3^+^CD8^+^PD-1^+^ T cell subpopulations by FlowSOM clustering. Among these ICs, HVEM had the highest co-expression level.

For the CD3^+^CD4^+^PD-1^+^ T cells of the study population, the raw t-SNE map is generated in Supplementary Figure S1A, and the expression levels of various ICs on the map are presented in Figure 4A. In comparison with other ICs, HVEM could be detected in a larger region (Figure 4A). On the t-SNE map, 14 subpopulations were clustered from CD3^+^CD4^+^PD-1^+^ T cells by the XShift module (Supplementary Figure S1B). A heatmap of the expression of 14 CD3^+^CD4^+^PD-1^+^ T cell subpopulations in 69 EOC cases is presented in Supplementary Figure S1C. Cluster X11 was the most common subpopulation (Figure 4B). Among the ICs, HVEM had the highest co-expression level in the 14 CD3^+^CD4^+^PD-1^+^ T cell subpopulations (Figure 4C). In addition, 14 subpopulations were clustered on the raw t-SNE map by FlowSOM (Supplementary Figure S1D). A heatmap of the expression of 14 CD3^+^CD4^+^PD-1^+^ T cell subpopulations in 69 EOC patients is demonstrated in Supplementary Figure S1E. Cluster F2 was the most common subpopulation (Figure 4D). Among the ICs, HVEM had the highest co-expression level in the 14 CD3^+^CD4^+^PD-1^+^ T cell subpopulations (Figure 4E).

For the CD3^+^CD8^+^PD-1^+^ T cells of the study population, the raw t-SNE map is developed in Supplementary Figure S1F, and the expression levels of various ICs on the map are presented in Figure 4F. Compared to other ICs, HVEM could be detected in a larger area (Figure 4F). On the t-SNE map, six subpopulations were clustered from CD3^+^CD8^+^PD-1^+^ T cells by the XShift module (Supplementary Figure S1G). A heatmap of the expression of six CD3^+^CD8^+^PD-1^+^ T cell subpopulations in 69 EOC cases is presented in Supplementary Figure S1H. Cluster X5 was the most common subpopulation (Figure 4G). Among the ICs, HVEM had the highest co-expression level in the six CD3^+^CD8^+^PD-1^+^ T cell subpopulations (Figure 4H). By FlowSOM, six subpopulations were also clustered on the raw t-SNE map (Supplementary Figure S1I). A heatmap of the expression of six CD3^+^CD8^+^PD-1^+^ T cell subpopulations in 69 EOC patients is shown in Supplementary Figure S1J. Cluster F6 was the most common subpopulation (Figure 4I). Among the ICs, HVEM had the highest co-expression level in the six CD3^+^CD8^+^PD-1^+^ T cell subpopulations (Figure 4J).

### 2.3. Addition of Anti-BTLA Ab to Other ICIs Can Generate More Potent Tumoricidal Effects

**Figure 5:**
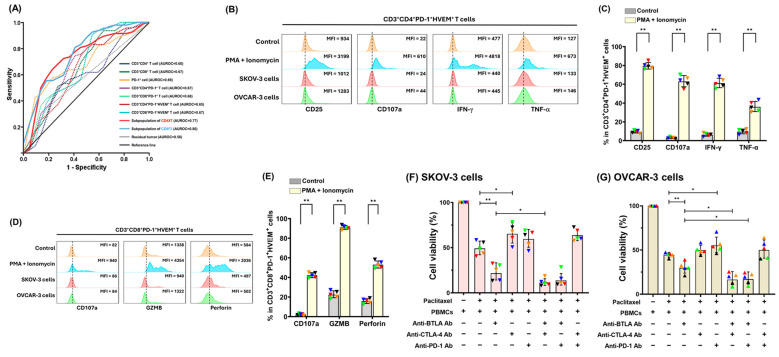
Role of PD-1^+^ T lymphocytes co-expressing HVEM. (**A**) ROC curves of various parameters for OS of EOC patients. (**B**) Representative histograms of activation markers in CD3^+^CD4^+^PD-1^+^HVEM^+^ T lymphocytes of PBMCs from healthy donors. Expression of CD25, CD107a, IFN-g, and TNF-a was up-regulated by PMA and ionomycin. (**C**) Bar graphs showing the percentages of activation markers in CD3^+^CD4^+^PD-1^+^HVEM^+^ T cells of PBMCs from healthy donors. (*n* = 5) (**D**) Representative histograms of activation markers in CD3^+^CD8^+^PD-1^+^HVEM^+^ T lymphocytes of PBMCs from healthy donors. Expression of CD107a, GZMB, and Perforin was up-regulated by PMA and ionomycin. (**E**) Bar graphs showing the percentages of activation markers in CD3^+^CD8^+^PD-1^+^HVEM^+^ T cells of PBMCs from healthy donors. (*n* = 5) (**F**) Killing assay of paclitaxel (PTX)-pretreated SKOV-3 cells. PBMCs combined with anti-BTLA Ab alone or anti-BTLA plus anti-CTLA-4 or anti-PD-1 Ab showed significant tumor-killing effects. (*n* = 5) (**G**) Killing assay of PTX-pretreated OVCAR-3 cells. PBMCs combined with anti-BTLA Ab alone or anti-BTLA plus anti-CTLA-4 or anti-PD-1 Ab showed significant tumor-killing effects. (*n* = 5) Note: * *p* < 0.05, ** *p* < 0.01, by Kruskal–Wallis test. (The colored triangles represent individual healthy donors.)

As shown in Figure 5A, the prediction potential for OS of 69 EOC patients by the subpopulation of CD3^+^CD4^+^PD-1^+^ T cells (CD4X7, area under the receiver operating characteristic [ROC] curve [AUROC] = 0.77) or CD3^+^CD8^+^PD-1^+^ T cells (CD8F2, AUROC = 0.80) was higher than that by other parameters. In addition, AUROC of more than 50% subpopulations of CD3^+^CD4^+^PD-1^+^ or more than 80% subgroups of CD3^+^CD8^+^PD-1^+^T cells was greater than that of residual tumor size (AUROC = 0.58, Figure 5A) (Supplementary Figure S2). Therefore, the PD-1^+^CD4^+^ andCD8^+^ T cells co-expressing HVEM could have a role to be further evaluated using PBMCs from the healthy donors.

Expression of CD25, CD107a, interferon-gamma (IFN-γ), or tumor necrosis factor-alpha (TNF-α) in the PD-1^+^HVEM^+^CD4^+^ T cells (Figure 5B,C) and expression of CD107a, Granzyme B (GZMB), or perforin in the PD-1^+^HVEM^+^CD8^+^ T cells (Figure 5D,E) were significantly higher with PMA and ionomycin stimulation than other conditions. The PD-1^+^HVEM^+^ CD4^+^ and CD8^+^ T cells could function as effector cells under the appropriate activation signals. When the checkpoint interaction was inhibited with ICIs, PBMCs combined with anti-BTLA Ab alone, or anti-BTLA plus anti-CTLA-4 or anti-PD-1 Ab showed the significant in vitro killing effects of PTX-pretreated SKOV-3 (Figure 5F) and OVCAR-3 (Figure 5G) cells.

**Figure 6:**
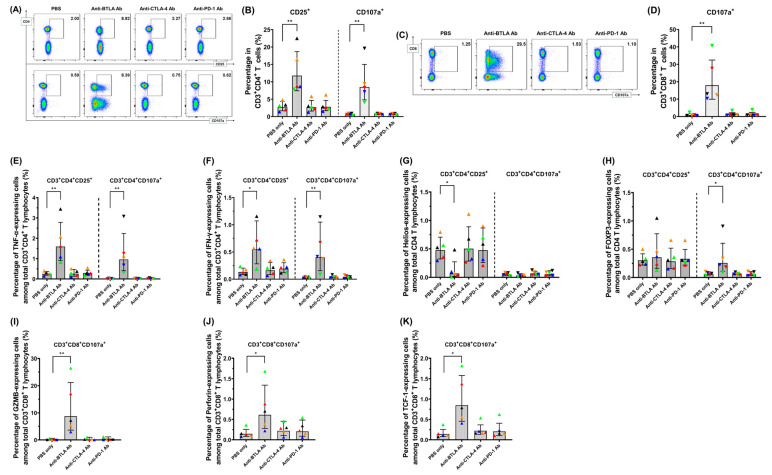
Potential of anti-BTLA Ab for activation of T lymphocytes. (**A**) Representative figures of percentage of CD25 and CD107a expressed on CD3^+^CD4^+^ T cells under various ICI treatments. (**B**) Bar graphs showing the percentages of activation markers on CD3^+^CD4^+^ T cells treated with various ICIs. The markers on CD3^+^CD4^+^ T cells increased in PBMCs under BTLA blockade. (*n* = 5) (**C**) Representative figures of percentage of CD107a expressed on CD3^+^CD8^+^ T cells under various ICI treatments. (**D**) Bar graphs showing the percentages of activation marker on CD3^+^CD8^+^ T cells treated with various ICIs. The marker on CD3^+^CD8^+^ T cells increased in PBMCs under BTLA blockade (*n* = 5). (**E**) Percentages of TNF-α-expressing CD3^+^CD4^+^CD25^+^ or CD3^+^CD4^+^CD107a^+^ cells in CD3^+^CD4^+^ T lymphocytes. TNF-α expression in the activated CD3^+^CD4^+^ T lymphocytes increased in PBMCs under BTLA blockade (*n* = 5). (**F**) Percentages of IFN-γ-expressing CD3^+^CD4^+^CD25^+^ or CD3^+^CD4^+^CD107a^+^ cells in CD3^+^CD4^+^ T lymphocytes. Among the various ICI treatment groups, IFN-γ expression in the activated CD3^+^CD4^+^ T lymphocytes increased in PBMCs under BTLA blockade (*n* = 5). (**G**) Percentages of Helios-expressing CD3^+^CD4^+^CD25^+^ or CD3^+^CD4^+^CD107a^+^ cells in CD3^+^CD4^+^ T lymphocytes. Helios expression in CD3^+^CD4^+^CD25^+^ T lymphocytes decreased under BTLA blockade (*n* = 5). (**H**) Percentages of FOXP3-expressing CD3^+^CD4^+^CD25^+^ or CD3^+^CD4^+^CD107a^+^ cells in CD3^+^CD4^+^ T lymphocytes. FOXP3 expression in CD3^+^CD4^+^ T lymphocytes increased in CD3^+^CD4^+^CD107a^+^ cells under BTLA blockade (*n* = 5). (**I**) Percentages of GZMB-expressing CD3^+^CD8^+^CD107a^+^ cells in CD3^+^CD8^+^ T lymphocytes. GZMB expression in the activated CD3^+^CD8^+^ T lymphocytes increased in PBMCs under BTLA blockade (*n* = 5). (**J**) Percentages of perforin-expressing CD3^+^CD8^+^CD107a^+^ cells in CD3^+^CD8^+^ T lymphocytes. Perforin expression in the activated CD3^+^CD8^+^ T lymphocytes increased in PBMCs under BTLA blockade (*n* = 5). (**K**) Percentages of TCF-1-expressing CD3^+^CD8^+^CD107a^+^ cells in CD3^+^CD8^+^ T lymphocytes. TCF-1 expression in activated CD3^+^CD8^+^ T lymphocytes increased in PBMCs under BTLA blockade (*n* = 5). Note: * *p* < 0.05, ** *p* < 0.01, by Kruskal–Wallis test. (The colored triangles represent individual healthy donors.)

Among the PBMCs from healthy donors treated with various ICIs, the activation markers, CD25 and CD107a on CD3^+^CD4^+^ T cells (Figure 6A,B) and the activation marker, CD107a on CD3^+^CD8^+^ T cells (Figure 6C,D) increased under BTLA blockade. Among the various ICI treatment groups, TNF-α (Figure 6E) and IFN-γ expression (Figure 6F) in CD3^+^CD4^+^CD25^+^ or CD3^+^CD4^+^CD107a^+^ activated T cells increased in CD3^+^CD4^+^ T cells under BTLA blockade. Helios expression decreased in CD3^+^CD4^+^CD25^+^ cells under BTLA inhibition (Figure 6G). Expression of forkhead box protein P3 (FOXP3, Figure 6H) increased in CD3^+^CD4^+^CD107a^+^ cells under BTLA inhibition. Among the various ICI treatment groups, GZMB (Figure 6I), perforin (Figure 6J), and T-cell factor 1 (TCF-1) (Figure 6K) expression in CD3^+^CD8^+^CD107a^+^-activated T cells were all increased under anti-BTLA Ab treatment. Consequently, blockade of BTLA in PBMCs resulted in driving T lymphocytes toward an activated phenotype.

## 3. Discussion

This study showed that percentages of PD-1^+^CD4^+^ T cells and PD-1^+^CD8^+^ T cells in the PBMCs of advanced EOC cases could positively correlate with DFS and OS. Co-expression of HVEM on these PD-1^+^ T lymphocytes was highly detected. In addition, the prediction potential for OS of EOC patients by the subpopulation of PD-1^+^CD4^+^ T cells or PD-1^+^CD8^+^ T cells was higher than that by other parameters. Under the appropriate activation signals, the PD-1^+^HVEM^+^ CD4^+^ and CD8^+^ T cells exhibited characteristics of effector cells. PBMCs combined with anti-BTLA plus anti-CTLA-4 or anti-PD-1 Ab had significant tumor-killing ability in vitro. Under the treatment of anti-BTLA Ab, T cells in the PBMCs drove toward an effector phenotype.

In the early development of some cancer sites, including EOCs, the tumor-associated antigens can be immunogenic [23,24,25]. However, the immune system ultimately no longer inhibits neoplastic cells after tumor immunogenicity editing in three phases: elimination, equilibrium, and escape [24]. Malignant diseases can be eradicated by the cytotoxicity produced by antigen-specific T lymphocytes in the elimination phase [24]. Occasionally, some tumor cell variants are not destroyed in the elimination phase and then enter the equilibrium stage. Because of immune selection pressure on these unstable cancer cells in equilibrium, they may enter the escape stage, in which anti-tumor immune responses fail to control diseases. Interaction between immune checkpoints is one mechanism underlying tumor outgrowth in the escape phase [26].

A study by Hamanish et al. has demonstrated that prognosis of patients with high expression of PD-L1 in ovarian cancer specimens was poorer than that of cases with a low PD-L1 expression level [12]. Low percentage of lymphocyte expression, PD-1, in PBMCs was associated with improved DFS and OS in the analysis by Chatterjee et al. However, 37 out of 63 EOC patients had advanced diseases, and the median follow-up period of 20.0 months was relatively short [27]. In our current study, all 69 EOCs were advanced diseases, and the median follow-up interval was 63.0 months (Supplementary Table S1). Patients with high percentages of PD-1^+^ T lymphocytes in PBMCs had favorable DFS and OC (Figure 3D–G). In addition, the prediction potential for OS of 69 cases by the subpopulation of PD-1^+^ T cells was higher than that by other parameters, including residual disease status after cytoreductive surgery, which is one of the most important prognostic factors for disease progression (Figure 5A and Supplementary Figure S2) [28,29,30].

In the tumor-bearing hosts, PD-1 expression on T lymphocytes in PBMCs is associated with an activated phenotype. These primed PD-1^+^ effector cells, which are present in the circulation of cancer patients, may have efficient abilities to eliminate tumor cells when they can be released from the interaction of PD-1/PD-L1 [31]. However, efficacy data and the results of clinical trials suggest that EOCs do not respond well to anti-PD-1/PD-L1 monotherapy [18]. The phenomenon can be explained by the fact that individual expression of PD-1 or other inhibitory ICs is not indicative of exhaustion, but co-expression of several inhibitory ICs is an essential manifestation. The higher the number of inhibitory ICs co-expressed on T lymphocytes, the more severe the exhaustion [21]. In our analysis, co-expression of HVEM on these PD-1^+^ T lymphocytes was highly detected (Figure 4), suggesting that the BTLA/HVEM axis may be a potential target for modulation in these T cell subsets. Consequently, simultaneous blockade of multiple inhibitory pathways can lead to great reversal of T cell exhaustion.

Based on the presence and distribution of associated T cells, tumors can be classified into hot, immunosuppressed, excluded, and cold groups. Except for hot tumors, complex pathways leading to tumor development are identified in other subtypes. Combinational strategies targeting different mechanism could be considered in the treatment of immunosuppressed tumors, including EOCs [32,33]. Our preclinical studies showed that ICIs combined with other agents, including chemotherapy, anti-angiogenic agents, and PARP inhibitors, can generate potent anti-tumor effects. Dual inhibition of HVEM/BTLA and PD-1/PD-L1 pathways can enhance the efficacy of PTX to treat tumors with intraperitoneal spread [22,34]. In this current study, PBMCs combined with anti-BTLA plus anti-CTLA-4 or anti-PD-1 Ab had significant tumor-killing effects on the PTX-pretreated tumor cells (Figure 5F,G) through activation of T cells by the BTLA blockade (Figure 6). Several completed phase II trials have also shown combinational strategies of ICIs, and other agents had beneficial results in EOC treatment [18].

Inhibitory checkpoint pathways in the suppressive TME act as an obstacle in the immune escape [8,35]. In addition to overcoming the immune suppression of checkpoint pathway using ICIs, another essential issue is to maintain and deliver the activated immune effector cells to the TME. In this current study, strategies of anti-HVEM-BTLA axis experimentally validated by PBMCs can have a potential role on the immunotherapy of EOCs. The ICIs are intravenously infused in clinical application, which can facilitate the selected agent to immediately and continuously interact with the target checkpoint axis on PBMCs. The possibility of transporting the immune cells with potent tumoricidal abilities to the TME is high.

Although our analysis suggested that investigation of IC profiling on PBMCs can reveal the ICs with prognostic and therapeutic potentials in EOC patients, several limitations were still noted in this study. First, the role of certain immune cells, such as CD3^+^CD56^+^ cells (natural killer T [NKT]-like cells), could not be well evaluated. The NKT-like cells comprise both classical and non-classical subsets and could be typically distinguished using TCRVα14 and CD14 [36]. Due to fluorescence channel constraints, these two molecules were not included in our flow cytometric panel, and therefore the subpopulations could not be adequately analyzed. Second, the PBMCs from ovarian cancer patients were preoperatively collected, and the sample volume of PBMCs was limited, making it difficult to further investigate T cell cytotoxicity levels and memory phenotypes. Third, in our in vitro tumor-killing assay, CTLA-4 and PD-1 blockade did not enhance PBMC cytotoxicity, likely due to simplified nature of the experimental system, which cannot reflect complex tumor–immune interactions in vivo. In contrast, the BTLA/HVEM blockade still promoted tumor cell killing, highlighting its therapeutic potential. Fourth, in our preliminary results, the role of BTLA blockade on T cells in human PMBCs was different from that in C57BL/6 mice. Lack of a humanized animal model incorporating human tumor cells and human immune cells prevents us from validating the therapeutic effects of combined chemotherapy and ICIs in vivo. Development of such a platform is needed to conduct future research. Fifth, the limited volume of healthy donor and patient samples restricted the analyses that could be performed, and therefore certain analyses were prioritized over others.

## 4. Materials and Methods

### 4.1. Patient and PBMC Preparation

A total of 69 women diagnosed with advanced EOCs were enrolled. The clinical records, therapeutic strategies, and disease follow-up protocols of these cases were described in our previous study [34]. The time from completion of the primary treatment until the date of verified tumor recurrence, progression, or last follow-up was considered as DFS. The interval from EOC diagnosis until the date of disease-related death or last visit was calculated as OS [37]. After the preoperative blood samples from EOC patients or blood samples from healthy donors were centrifugated at 1000× *g* for 10 min, PBMCs were isolated using Ficoll–Paque solution. PBMCs from 69 EOC patients were stored at −135 °C until analyses and those from 5 healthy donors were maintained in RPMI 1640 and 10% human serum at 37 °C in a 5% carbon dioxide atmosphere in experiments.

### 4.2. Surface and Intracellular Staining of PBMCs Analyzed by Flow Cytometry, t-SNE, XShift, and FlowSOM

Surface and intracellular markers of PBMCs were stained with various Abs (Supplementary Tables S2 and S3) as described previously [38,39]. Matched isotypes for each Ab were used as control. Data were collected by BD FACSLyric^TM^ and analyzed using FlowJo software (ver 10.8) (BD Biosciences, San Jose, CA, USA). Raw t-SNE maps were generated before data concatenation using the DownSample function of all samples. The t-SNE algorithm with XShift and FlowSOM modules was applied to cluster subpopulations of T lymphocytes as described in our previous study [22]. These identified clusters were correlated with patient survival.

### 4.3. Preparation of Cell Lines

Ovarian cancer cell lines SKOV-3 and OVACR-3 were purchased from ATCC (American Type Culture Collection, Manassas, VA, USA). SKOV-3 cells were maintained in McCoy’s 5a medium with 10% fetal bovine serum (FBS) at 37 °C in a 5% carbon dioxide atmosphere. OVCAR-3 cells were maintained in RPMI1640 with human recombinant insulin (4 mg/mL) and 20% FBS at 37 °C in a 5% carbon dioxide atmosphere.

### 4.4. Preparation of PTX and Anti-PD-1, CTLA-4, and BTLA Abs

Paclitaxel (PTX) (Sigma-Aldrich, St. Louis, MI, USA), anti-human CTLA-4 (Ipilimumab Biosimilar, Bio X cell, Lebanon, NH, USA), and PD-1 (Pembrolizumab Biosimilar, Bio X cell, Lebanon, NH, USA) Abs were purchased. PTX was initially dissolved in DMSO and subsequently diluted with PBS for experimental use. All antibodies were stored in PBS (pH 7.0) and diluted in the same buffer prior to use. The anti-BTLA neutralizing Ab was generated to block the BTLA-HVEM axis. Based on the binding sequence of BTLA and HVEM [40], a synthesized peptide ESCDVQLYIKRQSE was used as the immunogen. Rabbits were immunized with this peptide, and serum was collected. The anti-human BTLA neutralizing Abs were then purified by affinity chromatography (GeneDireX, Inc., Taoyuan, Taiwan).

### 4.5. Activation Potential of PD-1^+^HVEM^+^ T Cells in PBMCs from Healthy Donors

PBMCs (5 × 10^5^ cells/mL) were seeded in 6-well plates and stimulated with phorbol myristate acetate (PMA, 100 ng/mL; dissolved in DMSO) and ionomycin (1 μg/mL; dissolved in DMSO), SKOV-3 cells (5 × 10^4^ cells/mL), OVCAR-3 cells (5 × 10^4^ cells/mL), or PBS (control group) for 24 h. Brefeldin A (1 μg/mL) was added to each well 4 h before analysis. The activation markers of CD3^+^CD4^+^ and CD3^+^CD8^+^ T lymphocytes are listed in Supplementary Table S3 (A,B), respectively [22]. All cells were then collected, stained, and analyzed for activation markers of PD-1^+^HVEM^+^ CD4^+^ and CD8^+^ T cells.

### 4.6. Tumoricidal Abilities of PBMCs from the Healthy Donors Treated with Various ICI Strategies

SKOV-3 and OVCAR-3 cells were seeded into 24-well plates (2 × 10^4^ cells/well) and pretreated with PTX (1 μg/mL) for 24 h. Then, PBMCs (2 × 10^5^ cells/well) were co-cultured with the tumor cells. Anti-BTLA (20 μg/mL), anti-CTLA-4 (20 μg/mL), and anti-PD-1 (20 μg/mL) Abs were added either in a single or in a combinational manner. PBS was used as negative control. After 20 h of incubation, the medium and suspended cells were removed and washed with PBS. The remaining tumor cells were then incubated with MTT reagent for 4 h. Cell viability was determined by measuring the OD value at 450 nm using VersaMax Microplate Reader (Molecular Devices, San Jose, CA, USA).

### 4.7. Impact of Respective ICIs on Activation of T Cells in PMBCs from Healthy Donors

Anti-BTLA (20 μg/mL), anti-CTLA-4 (20 μg/mL), and anti-PD-1 (20 μg/mL) Abs were cocultured with PBMCs from healthy donors for 24 h. PBS was used as negative control. Brefeldin A (1 μg/mL) was added to each well 4 h before analysis. All cells were then stained and analyzed for activation markers of CD4^+^ and CD8^+^ T cells (Supplementary Table S3). Helios expression can ensure stable suppressive activities of regulatory T cells (Tregs) [41]. TCF-1 was related to exhaustion of cytotoxic T cells [42].

### 4.8. Statistical Analyses

All statistical analyses were performed in SPSS for Windows version 15.0 (SPSS Inc., Chicago, IL, USA). The data were expressed as geometric mean ± geometric standard deviation factor. The 25th percentile was derived from the lowest quartile of all patient samples for the percentage of each immune cell population or expression level of each immune checkpoint by ranking the data and taking the value at the cumulative 25% position. This cutoff was chosen because the study aimed to identify molecules with potential as therapeutic targets which required a minimum level of expression. The association between expression level of respective immune cell and patient survival was assessed using the Kaplan–Meier method. Residual disease status after cytoreductive surgery has been considered as one of the most important prognostic factors for disease progression of advanced EOCs [28,29]. The survival analyses were adjusted for this confounding factor. The results of in vitro experiments using PBMCs from the healthy donors were examined by Kruskal–Wallis test. *p* < 0.05 was defined as significant. Spearman’s rank correlation was applied to evaluate the relationship various immunocytes and PD-1-expressing cells in PBMCs. The correlation coefficient, R value ≥ 0.4, was defined as moderately correlated [43,44]. Sensitivity and specificity of various parameters for OS of EOC patients were estimated using the ROC curves. We calculated the AUROC of various parameters [45].

## 5. Conclusions

In conclusion, percentages of PD-1^+^CD4^+^ T cells and PD-1^+^CD8^+^ T cells in the PBMCs of advanced EOC cases could positively correlate with DFS and OS. Co-expression of HVEM on these PD-1^+^ T lymphocytes was highly detected. Tumoricidal ability of the PBMCs treated with addition of anti-BTLA Ab to other ICIs, including anti-PD-1 Ab, was more potent than that of PBMCs receiving single ICI treatment. Therefore, inhibition of the HVEM-BTLA axis may be considered as a potential solution to the current puzzle of ICI monotherapy for EOCs.

## Data Availability

Data are contained within the article and Supplementary Materials.

## Supplementary Materials

The following supporting information can be downloaded at: https://www.mdpi.com/article/10.3390/ph18091295/s1, Table S1: Clinicopathologic characteristics of study population (69 cases); Table S2: Antibodies for flowcytometry; Table S3: Activation markers of T lymphocytes. Figure S1: T cell subpopulations on t-SNE maps and their distribution across individual patients; Figure S2: ROC curves of various T cell subpopulations for OS of EOC patients.

## Abbreviations

The following abbreviations are used in this manuscript:

EOC	epithelial ovarian cancer
PTX	paclitaxel
PARP	poly-ADP ribose polymerase
ICIs	immune checkpoint inhibitors
CTLA-4	cytotoxic T lymphocyte antigen 4
PD-1	programmed cell death 1
PD-L1	programmed death-ligand 1
ICs	immune checkpoints
TME	tumor microenvironment
PBMCs	peripheral blood mononuclear cells
t-SNE	t-distributed stochastic neighbor embedding
Abs	antibodies
DFS	disease-free survival
OS	overall survival
FBS	fetal bovine serum
BTLA	B and T lymphocyte attenuator
HVEM	herpes virus entry mediator
PMA	phorbol myristate acetate
Tregs	regulatory T cells
TCF-1	T-cell factor 1
ROC curve	receiver operating characteristic curve
AUROC	area under the ROC curve
TIM-3	T-cell immunoglobulin domain and mucin domain 3
GZMB	granzyme B
