# Supplementary material for: Analysis of Immune Checkpoints on Peripheral Blood Mononuclear Cells Can Predict Clinical Outcome and Reveal Potential of HVEM-BTLA Axis in Epithelial Ovarian Cancers
Source: Pharmaceuticals (Basel). 2025 Aug 29;18(9):1295. doi: 10.3390/ph18091295 (PMC12472872; doi:10.3390/ph18091295)
Supplement: Supplementary file 1 [file pharmaceuticals-18-01295-s001.zip › Supplementary Table S1.pdf]

Supplementary Table S1. Clinicopathologic characteristics of study population (69 cases)

**Age** (years) (median, range)	58.0 (32.0-85.0)
**Follow-up period** (months) (median, range)	63.0 (12.0-154.0)
**Pre-operative CA-125** (U/mL) (median, range)	1198.6 (33.5-27961.5)
**BMI** (kg/m2) (median, range)	23.0 (17.7-38.8)
	**Patient number**
**FIGO stage**	
III	58
IV	11
**Tumor Histology**	
Serous	61
Non-serous	8
**Primary treatment***	
PDS + postoperative C/T	60
Neoadjuvant C/T + IDS + postoperative C/T	9
**Postoperative residual tumor#**	
≤ 1cm	49
> 1cm	20
**Disease recurrence**	
Yes	54
No	15
**Patient status**	
Disease-related death	22
Alive	47

CA-125, cancer antigen 125; BMI, body mass index; FIGO, the International Federation of Gynecology and Obstetrics; PDS, primary debulking (cytoreductive) surgery; C/T, chemotherapy; IDS, interval debulking (cytoreductive) surgery; +, and

*Chemotherapeutic regimens included platinum-based chemotherapy and platinum-based chemotherapy combined with bevacizumab.

#Residual tumor status was recorded after debulking (cytoreductive) surgery.
