# Supplementary material for: Analysis of Immune Checkpoints on Peripheral Blood Mononuclear Cells Can Predict Clinical Outcome and Reveal Potential of HVEM-BTLA Axis in Epithelial Ovarian Cancers
Source: Pharmaceuticals (Basel). 2025 Aug 29;18(9):1295. doi: 10.3390/ph18091295 (PMC12472872; doi:10.3390/ph18091295)
Supplement: Supplementary file 1 [file pharmaceuticals-18-01295-s001.zip › Supplementary Table S2.pdf]

Supplementary Table S2. Antibodies for flowcytometry

Specificity	Fluorochrome	Clone	Company
CD3	peridinin chlorophyll protein (PerCP)-Cy5.5	UCHT1	BD Phamingen™
CD4	brilliant violet (BV) 786	SK3	BD Horizon™
CD8	BV605	SK1	BD Horizon™
CD19	allophycocyanin (APC)-Cy7	HIB19	BD Horizon™
CD56	phycoerythrin (PE)-Cy7	B159	BD Phamingen™
CD1a	BV510	HI149	BD Phamingen™
BTLA	APC	J168-540	BD Phamingen™
CTLA-4	R718	BNI3	BD Horizon™
HVEM	BV421	CW10	BD Horizon™
PD-1	BV711	EH12.1	BD Horizon™
PD-L1	BB515	MIH1	BD Horizon™
TIM-3	PE	7D3	BD Horizon™
