# Supplementary material for: Analysis of Immune Checkpoints on Peripheral Blood Mononuclear Cells Can Predict Clinical Outcome and Reveal Potential of HVEM-BTLA Axis in Epithelial Ovarian Cancers
Source: Pharmaceuticals (Basel). 2025 Aug 29;18(9):1295. doi: 10.3390/ph18091295 (PMC12472872; doi:10.3390/ph18091295)
Supplement: Supplementary file 1 [file pharmaceuticals-18-01295-s001.zip › Supplementary Table S3.pdf]

**Supplementary Table S3. Activation markers of T lymphocytes**

| A. Activation markers of $CD4^+$ T lymphocytes | | | |
|---|---|---|---|
| **Specificity** | **Fluorochrome** | **Clone** | **Company** |
| CD3 | PerCP-Cy5.5 | UCHT1 | BD Phamingen™ |
| CD4 | BV786 | SK3 | BD Horizon™ |
| IFN-γ | BV605 | B27 | BD Horizon™ |
| CD107a | APC-H7 | H4A3 | BD Phamingen™ |
| TNF-α | RB780 | MAb11 | BD Horizon™ |
| CD25 | BV510 | M-A251 | BD Horizon™ |
| FOXP3 | BB515 | 259D/C7 | BD Phamingen™ |
| Helios | PE | 22F6 | BD Phamingen™ |
| HVEM | BV421 | CW10 | BD Horizon™ |
| PD-1 | BV711 | EH12.1 | BD Horizon™ |
| **B. Activation markers of $CD8^+$ T lymphocytes** | | | |
| **Specificity** | **Fluorochrome** | **Clone** | **Company** |
| CD3 | PerCP-Cy5.5 | UCHT1 | BD Phamingen™ |
| CD8 | BV605 | SK1 | BD Horizon™ |
| Perforin | PE-Cy7 | B-D48 | BioLegend |
| CD107a | APC-H7 | H4A3 | BD Phamingen™ |
| HVEM | BV421 | CW10 | BD Horizon™ |
| PD-1 | BV711 | EH12.1 | BD Horizon™ |
| TCF-1 | PE | S33-966 | BD Phamingen™ |
| Granzyme B | BV510 | GB11 | BD Horizon™ |
