# Supplementary figures and images for: Analysis of Immune Checkpoints on Peripheral Blood Mononuclear Cells Can Predict Clinical Outcome and Reveal Potential of HVEM-BTLA Axis in Epithelial Ovarian Cancers
Source: Pharmaceuticals (Basel). 2025 Aug 29;18(9):1295. doi: 10.3390/ph18091295 (PMC12472872; doi:10.3390/ph18091295)

## Slide 1
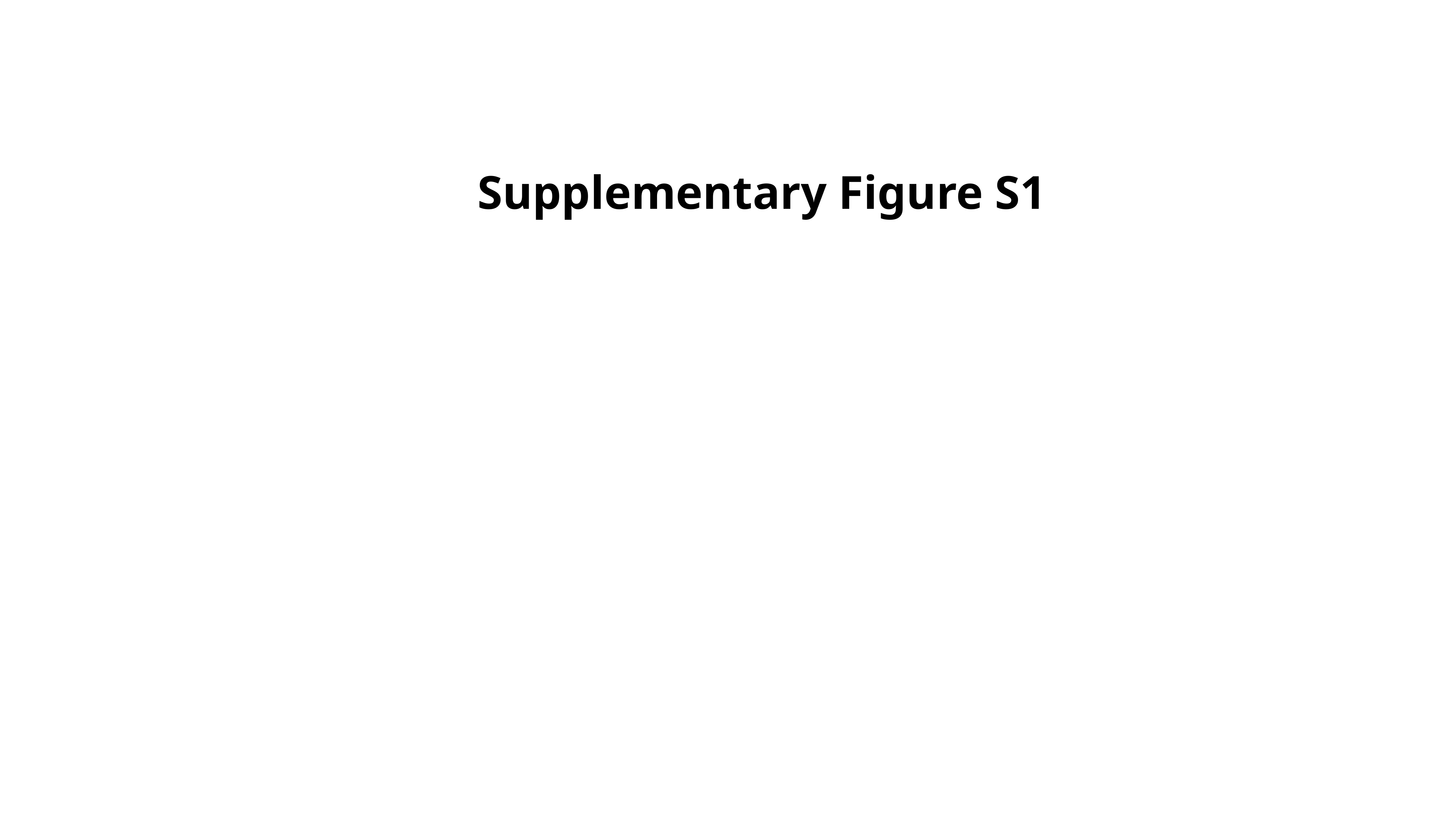

Supplementary Figure S1

## Slide 2
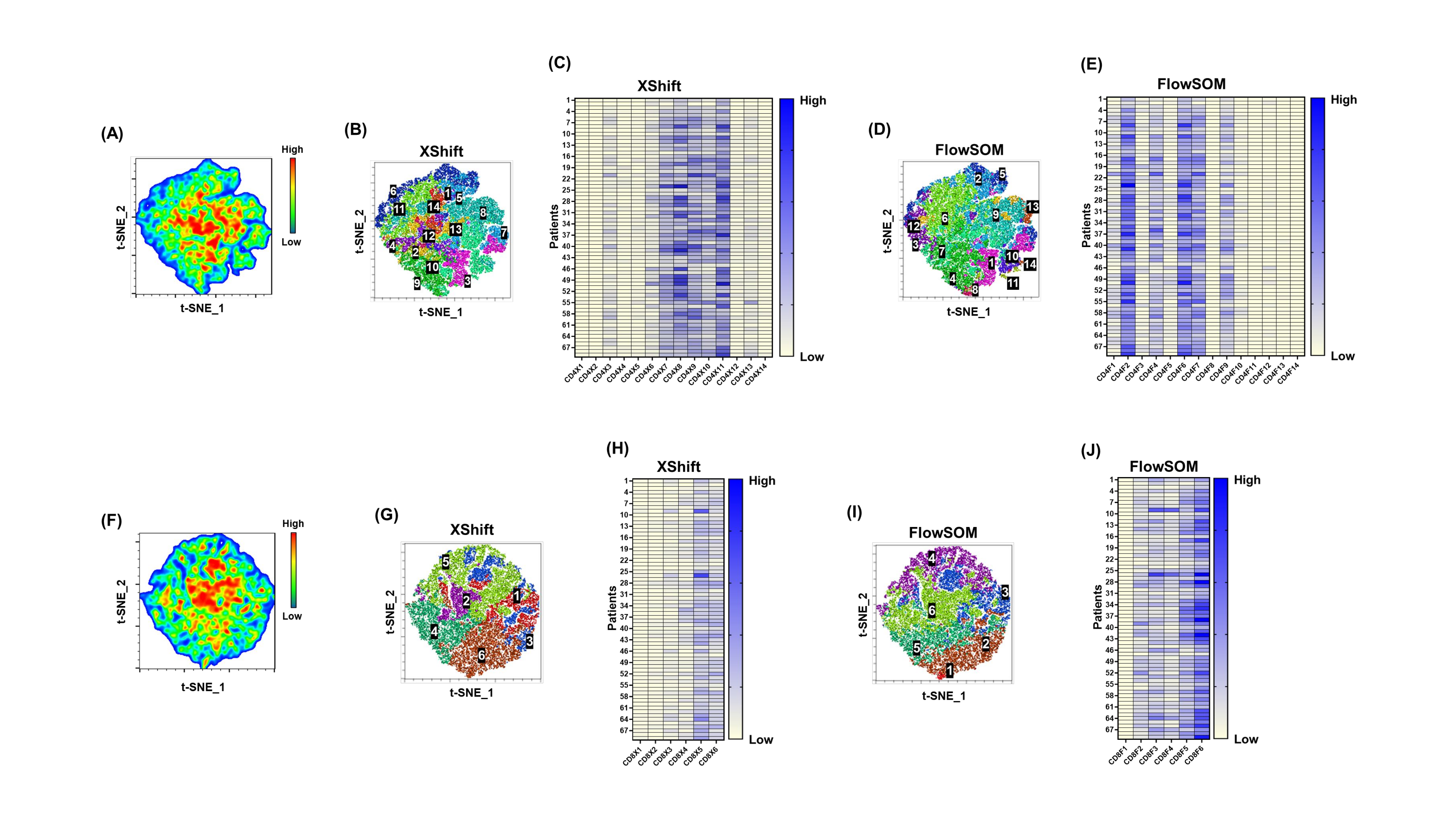

Supplement: Supplementary file 1 [file pharmaceuticals-18-01295-s001.zip › Supplementary Figure S1.pptx]

## Slide 1
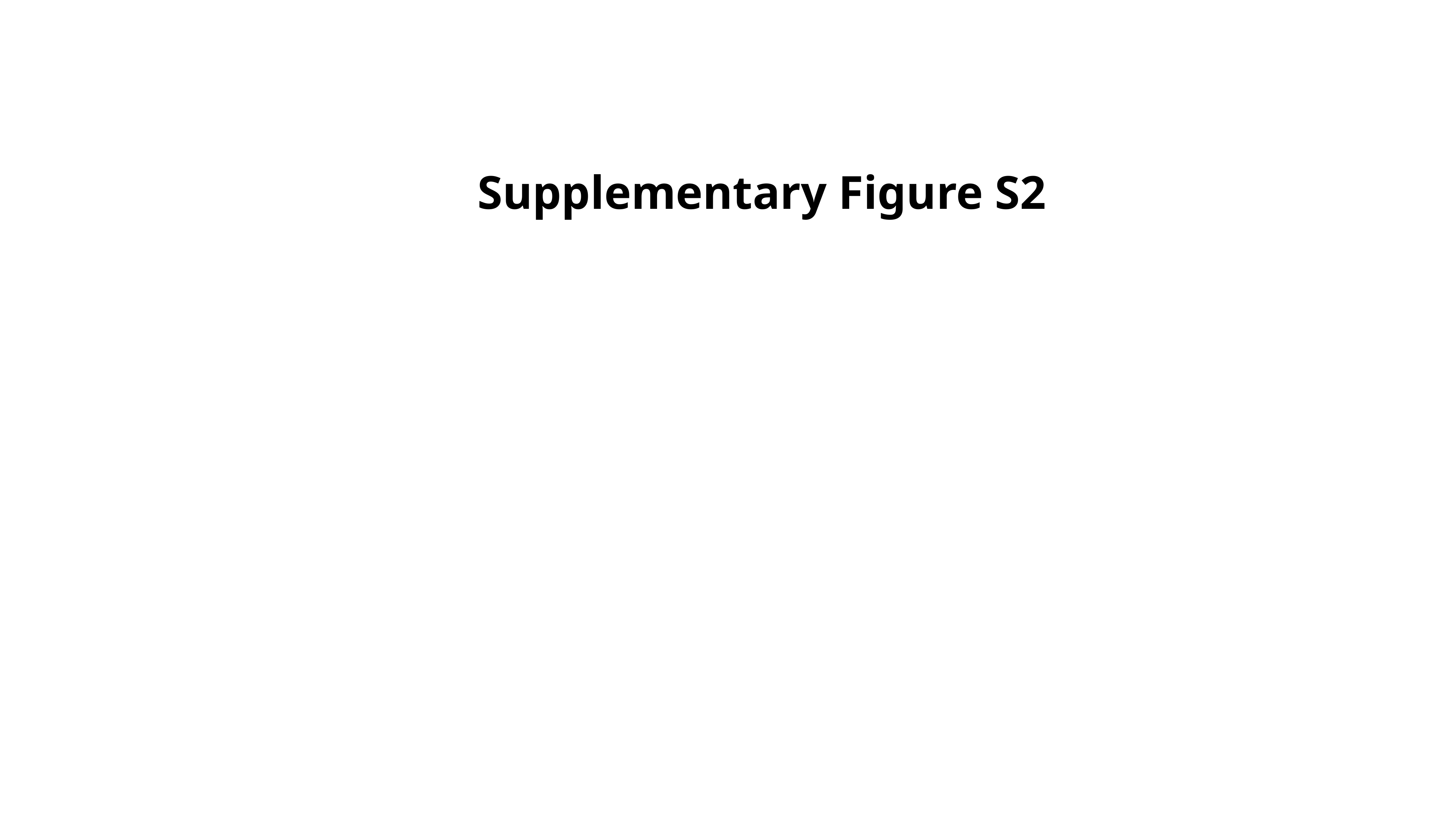

Supplementary Figure S2

## Slide 2
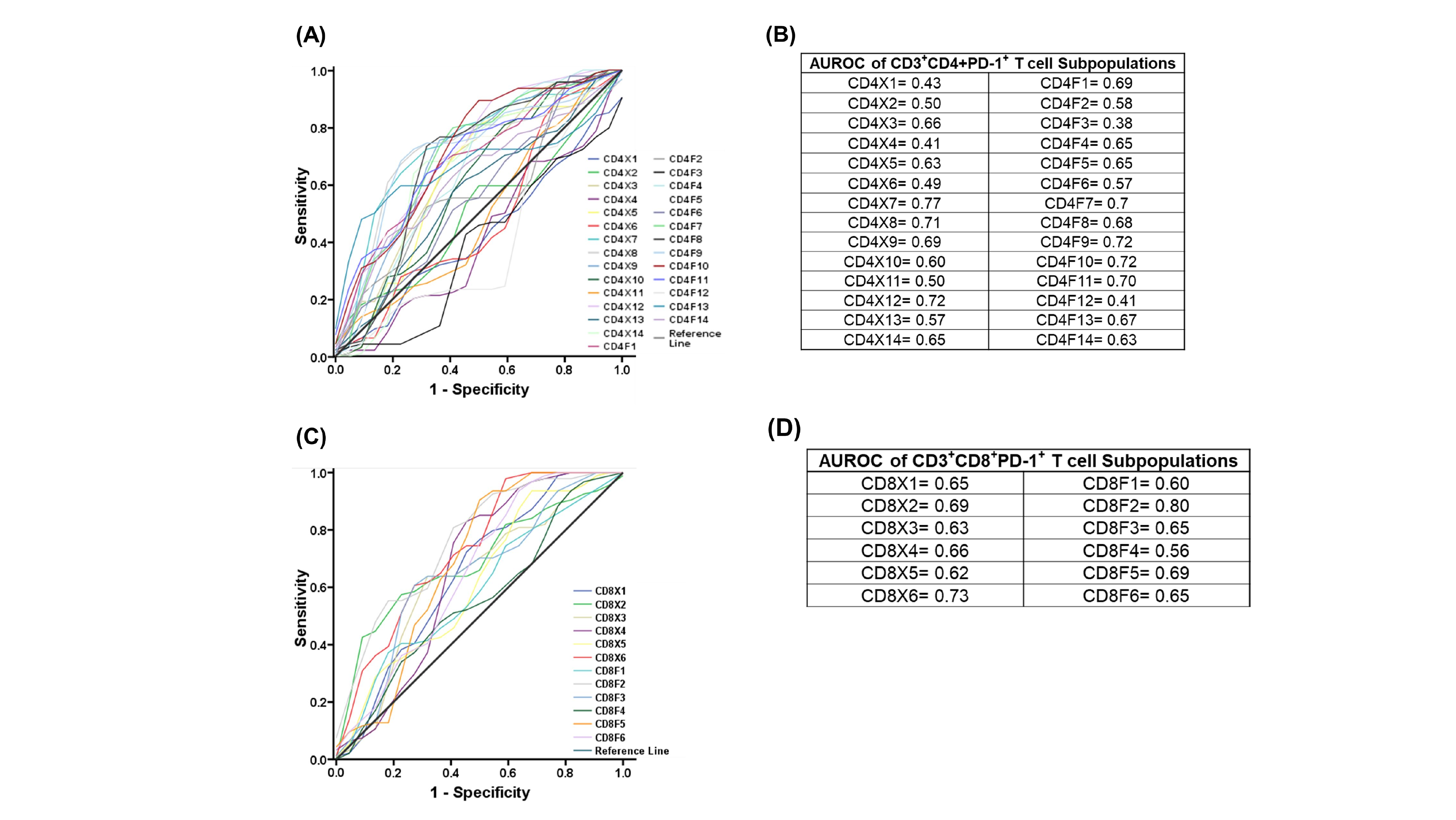

Supplement: Supplementary file 1 [file pharmaceuticals-18-01295-s001.zip › Supplementary Figure S2.pptx]
